# Supplementary material for: Dysbiosis of the Gut Microbiota and Kynurenine (Kyn) Pathway Activity as Potential Biomarkers in Patients with Major Depressive Disorder
Source: Nutrients. 2023 Apr 3;15(7):1752. doi: 10.3390/nu15071752 (PMC10096701; doi:10.3390/nu15071752)
Supplement: Supplementary file 1 [file nutrients-15-01752-s001.zip › Supplementary Table S1.pdf]

Table S1. Corrected p-value of the differences of functional genes between MDD patients and control

Name	Fold Change(F/GUT)	Corrected *p*-value (<0.05)	Description Function
S	1.035	1.476E-05	Function unknown
E	1.023	0.001102	Amino acid transport and metabolism
G	1.098	2.45E-06	Carbohydrate transport and metabolism
J	0.896	2.234E-08	Translation, ribosomal structure and biogenesis
M	0.950	0.001102	Cell wall/membrane/envelope biogenesis
L	0.962	0.0002023	Replication, recombination and repair
P	1.059	0.003702	Inorganic ion transport and metabolism
C	1.040	1.063E-05	Energy production and conversion
H	0.957	0.007206	Coenzyme transport and metabolism
F	0.944	7.308E-05	Nucleotide transport and metabolism
D	0.926	2.45E-06	Cell cycle control, cell division, chromosome partitioning
N	0.806	0.0002023	Cell motility
A	3.428	1.476E-05	RNA processing and modification
