# Supplementary figures and images for: Dysbiosis of the Gut Microbiota and Kynurenine (Kyn) Pathway Activity as Potential Biomarkers in Patients with Major Depressive Disorder
Source: Nutrients. 2023 Apr 3;15(7):1752. doi: 10.3390/nu15071752 (PMC10096701; doi:10.3390/nu15071752)

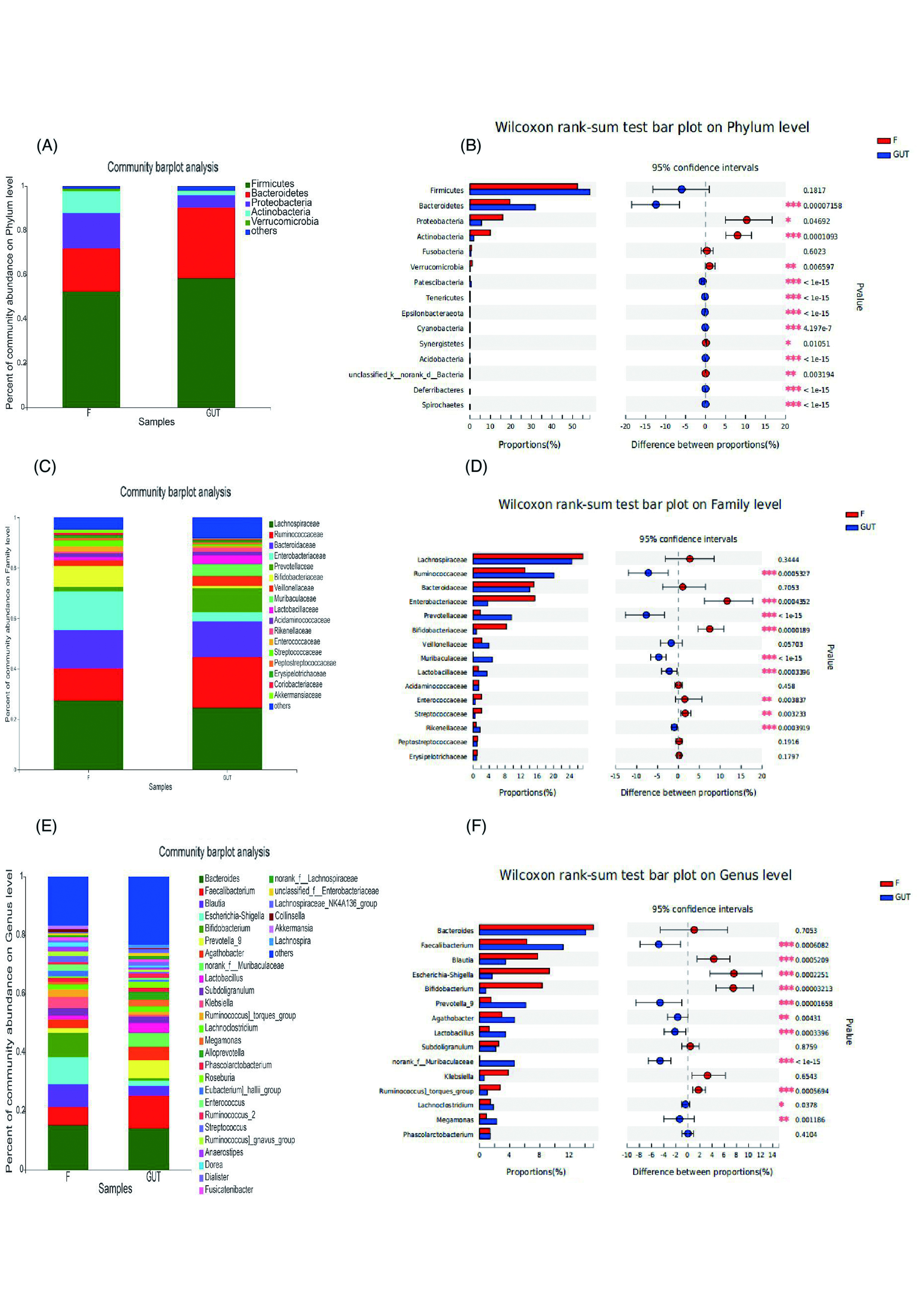

Supplement: Supplementary file 1 [file nutrients-15-01752-s001.zip › Supplementary Figure S1.jpeg]
